# High-Density Lipoprotein Predicts Intrahospital Mortality in Influenza

**DOI:** 10.3390/jcm13237242

**Published:** 2024-11-28

**Authors:** Matthias Wolfgang Heinzl, Markus Freudenthaler, Paul Fellinger, Lisa Kolenchery, Michael Resl, Carmen Klammer, Florian Obendorf, Lukas Schinagl, Thomas Berger, Margot Egger, Benjamin Dieplinger, Martin Clodi

**Affiliations:** 1 Department of Internal Medicine, Konventhospital Barmherzige Brueder Linz (St. John of God Hospital Linz), 4020 Linz, Austria; 2 CICMR—Clinical Institute for Cardiovascular and Metabolic Research, Johannes Kepler Universität Linz (JKU Linz), 4040 Linz, Austria; 3 Department of Laboratory Medicine, Konventhospital Barmherzige Brueder Linz and Ordensklinikum Linz, 4020 Linz, Austria; 4 Division of Endocrinology and Metabolism, Department of Medicine III, Medical University of Vienna, 1090 Wien, Austria; 5 Medical Faculty, Johannes Kepler University Linz, 4040 Linz, Austria

**Keywords:** influenza, acute viral infection, lipoproteins, HDL, lipid scavenging, anti-inflammatory

## Abstract

**Background:** Although it is known that high-density lipoprotein (HDL) exerts important anti-inflammatory effects and that low HDL plasma concentrations represent a negative prognostic marker in bacterial infections and sepsis, not much is known about possible implications of HDL in acute viral infections such as influenza. **Methods:** We performed a retrospective, single-centre analysis of influenza patients hospitalised during the 2018/19 and 2019/20 influenza seasons and analysed the impact of HDL concentrations on inflammation and mortality. **Results**: 199 influenza patients (173 male patients) were admitted during the 2018/19 and 2019/20 influenza seasons with a mortality rate of 4.5%. HDL was significantly lower in deceased patients (median HDL 21 (IQR 19–25) vs. 35 (IQR 28–44) mg/dL; *p* = 0.005). Low HDL correlated with increased inflammation and HDL was an independent negative predictor regarding mortality after correction for age and the number of comorbidities both overall (OR = 0.890; *p* = 0.008) and in male patients only (OR = 0.891; *p* = 0.009). **Conclusions:** Low HDL upon hospital admission is associated with increased inflammation and is an independent predictor for increased mortality in male patients with influenza A.

## 1. Introduction

Numerous interactions between components of the lipid metabolism, especially high-density lipoprotein (HDL), and infections have been described. Although high plasma concentrations of low-density lipoprotein (LDL) as a main risk factor for atherosclerotic cardiovascular disease and cardiovascular mortality is largely undisputed [1,2], lipoproteins also seem to exert complex and protective effects in severe infections. In elderly patients [3,4], as well as patients requiring haemodialysis [5], low LDL and HDL plasma concentrations appear to be associated with increased all-cause mortality.

In an experimental setting, such protective effects have been shown by numerous studies, especially for HDL, which is associated with reduced inflammatory response to infective stimuli [6,7,8]. One of the mechanisms of the protective effects of lipoproteins in bacterial infection has been identified as the binding and “scavenging” of pathogen lipids, especially endotoxin (lipopolysaccharide (LPS) of Gram-negative bacteria) and lipoteichoic acid (LTA) of Gram-positive bacteria, leading to hepatobiliary excretion and thus reduced inflammatory response [9].

Low concentrations of HDL are associated with increased inflammatory response upon exposure to LPS in the Human Endotoxin Model [8,10], and very low HDL is associated with an increased risk of infectious disease [11,12].

In clinical studies, the negative prognostic impact of low lipoprotein levels in infections has mainly been shown in ICU patients with sepsis and microbiological proof of bacterial infection [13,14,15]. In severe bacterial sepsis, low HDL (<20 mg/dL) at the time of hospital admission was shown to be a marker of increased mortality, and low HDL is associated with an increased inflammatory response [15], whereas another study found an HDL < 25.1 mg/dL to be a threshold for impaired prognosis in a sepsis cohort [16].

It remains unclear, however, whether concentrations of lipoproteins upon hospitalisation also represent a prognostic factor in infections apart from in severe bacterial infections in ICU patients and sepsis and especially in common acute viral infections such as influenza.

## 2. Methods

This study was performed to investigate concentrations of lipoproteins, especially HDL, and possible associations with markers of inflammation and outcome in influenza patients hospitalised at our institution during the 2018/19 and 2019/20 influenza seasons. Additionally, we compared data of patient characteristics and outcome with those from previously published data from the 2017/18 influenza season at our institution [17].

The study was conducted as a retrospective analysis. The St. John of God Hospital Linz is situated in the city centre of Linz, Upper Austria. The study was performed in accordance with the Declaration of Helsinki and International Conference on Harmonization Good Clinical Practice (ICH-GCP) guidelines and was approved by the responsible local ethics committee (Gemeinsame Ethikkommission der Barmherzigen Schwestern und Barmherzige Brüder Linz; 14 May 2018; EKB 07/18). Data were anonymised, and access to the data was restricted to authorised personnel.

Hospitalised patients who tested positive for influenza by nasopharyngeal swab were included in the study. Nasopharyngeal swabs were collected from all patients presenting with influenza-like illness and tested for influenza A and influenza B by using the Xpert^®^ Xpress Flu-RSV assay (Gene Xpert, Cepheid, Sunnyvale, CA, USA) at our central laboratory. The RT-PCR assay procedures were performed according to the manufacturer’s recommended protocol. Routine parameters such as C-reactive protein (CRP), serum creatinine (enzymatic method), lipid parameters including HDL, high-sensitivity cardiac troponin I (hs-cTnI), and B-type natriuretic peptide (BNP) were measured in heparin-treated plasma with standard assays on an Architect c16000 analyser (Abbott Diagnostics, Hong Kong), and glycated haemoglobin (HbA1c) was analysed on the HA-8180T (A. Menarini diagnostics, Hong Kong), respectively. Estimated glomerular filtration rate (eGFR) was calculated using the Modification of Diet in Renal Disease (MDRD) GFR equation.

Initial CRP, creatinine, eGFR, and glucose were determined during the first blood draw in the emergency department. Initial troponin and BNP values, HDL, LDL, triglycerides, and total cholesterol were measured either in the emergency department or within the first 48 h upon admission. Later measurements were not considered for baseline values. Stress hyperglycaemia ratio (SHR) was calculated with the formula SHR = [admission blood glucose (mg/dL)/18]/[1.59 × HbA1c (%) − 2.59] [18].

The neuraminidase inhibitor oseltamivir, as well as antibiotics, was administered as deemed indicated by the treating physician.

Exclusion criteria were those aged under 18 years and with a missing medical history or clinical examination. For statistical analysis, comorbidities (diabetes mellitus, arterial hypertension, kidney disease, coronary heart disease, COPD, and malignant disease) were identified by obtaining patients’ medical histories as well as diagnoses during hospital stay.

Both the collection and recording of data and the analysis of descriptive statistics were carried out using Microsoft^®^ Excel^®^. Statistical analysis including logistic regression analysis and receiver operator characteristic (ROC) analysis was performed using IBM^®^ SPSS27^®^ and SPSS29^®^ and GraphPad Prism (GraphPad Software, Version 10, San Diego, CA, USA). This was an exploratory study. For statistical calculations, values below the threshold of detection were set to the threshold of detection.

## 3. Results

### 3.1. Patient Characteristics

A total of 199 patients with positive tests for influenza were admitted to our institution during the 2018/19 and the 2019/20 influenza seasons. The median age and interquartile range (IQR) were 70 (56–80) years, and the median BMI was 26.2 (23.8–29.8).

As indicated in Table 1 and Table 2, there was an important disproportion between female and male patients, which is due to the structural organisation of hospital beds in the city of Linz. All of the 26 female inpatients survived, and only one was treated at the ICU.

Nine patients died, all of whom were male. Of the deceased patients, 7 were treated at the ICU. The two deceased patients who were not treated at the ICU were well beyond 80 years of age and in poor general condition. The deceased patients were between 59 and 86 years old (median age was 72 (IQR 70–84) years), and all but one had at least two comorbidities. The remaining deceased patient without formally diagnosed comorbidities was 86 years old.

There was an important and significant difference between women and men (median HDL values were 46 (IQR 35–69) mg/dL for women and 33 (IQR 26–43) mg/dL for men; *p* = 0.004 as calculated by Mann–Whitney U-test). Due to this imbalance in HDL values between men and women and the very small number of female patients, further calculations beyond descriptive statistics were thus only performed for male patients. However, logistic regression for HDL and a comparison of HDL values between deceased patients and survivors were additionally performed for all patients. The reported data were calculated for male patients unless otherwise stated.

### 3.2. Low HDL Is a Significant Predictor for Death

A comparison between HDL plasma concentrations among patients who survived vs. deceased patients revealed markedly and significantly higher HDL among survivors (median HDL in male patients was 34 (IQR 27–43) mg/dL in survivors vs. 21 (19–25) mg/dL in deceased male patients; *p* = 0.007 as calculated for male patients by Mann–Whitney U-test). This difference was even more pronounced when calculated for all patients instead of male patients only (with a median HDL of 35 (IQR 28–44) vs. 21 (19–25) mg/dL; *p* = 0.005).

**Figure 1:**
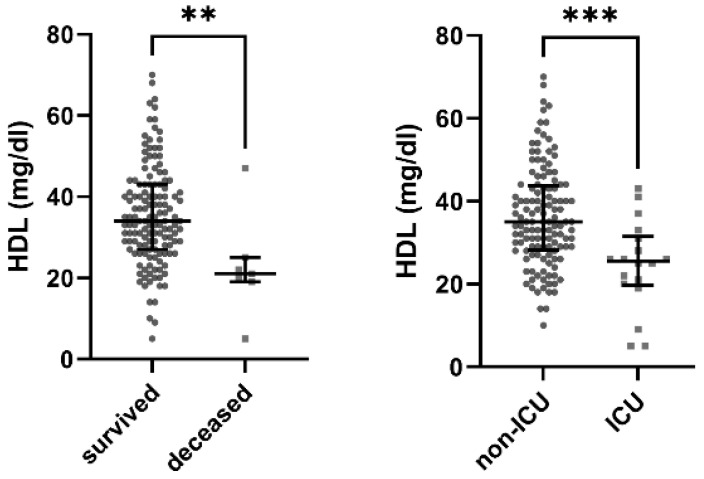
Boxplot showing HDL concentrations in male hospitalised influenza patients upon admission. HDL was significantly higher in patients who survived vs. deceased patients as well as in non-ICU vs. ICU patients. Median initial HDL was 34 (IQR 27–43) mg/dL in survivors vs. 21 (IQR 19–25) mg/dL in deceased male patients (** *p* = 0.007 as calculated by Mann–Whitney U-test). Median initial HDL was 35 (IQR 29–44) mg/dL in male non-ICU patients vs. 26 (20–31 mg/dL) in male ICU patients (*** *p* < 0.001 as calculated by Mann–Whitney U-test). Results were similar when testing all patients including females.

Similarly, initial HDL in patients admitted to the ICU was significantly lower compared to non-ICU patients (median initial 26 (IQR 20–31) mg/dL vs. 35 (IQR 29–44) mg/dL in male patients and 26 (IQR 20–33) vs. 35 (IQR 29–44) mg/dL in all patients; *p* < 0.001 as calculated for both by Mann–Whitney U-test); see Figure 1. All but one of the deceased patients had HDL values ≤ 25 mg/dL.

In a logistic regression model that included age, the number of comorbidities (diabetes mellitus, arterial hypertension, kidney disease, coronary heart disease, COPD, and malignant disease) and HDL, neither the patients’ age (OR = 1.031; *p* = 0.397) nor the number of comorbidities (OR = 0.846, *p* = 0.623) was a significant predictor for death. Low HDL concentrations, however, proved to be a significant predictor of intrahospital mortality after correction for age and the number of comorbidities (OR = 0.891, *p* = 0.009 for male patients; OR = 0.89, *p* = 0.008 for all patients).

When metric HDL values were divided into groups (0–10; 10–20; 20–30; 30–40; 40–50; 50–60; 60–70; 70–80 mg/dL), the effect of HDL as a marker for prognosis after correction for age and the number of comorbidities became more pronounced (OR = 0.427; *p* = 0.001 for male patients).

Furthermore, HDL also proved to be a significant negative predictor for ICU admission in logistic regression corrected for age and the number of comorbidities (OR = 0.911; *p* = 0.001 for male patients; OR = 0.920; *p* = 0.002 for all patients).

**Figure 2:**
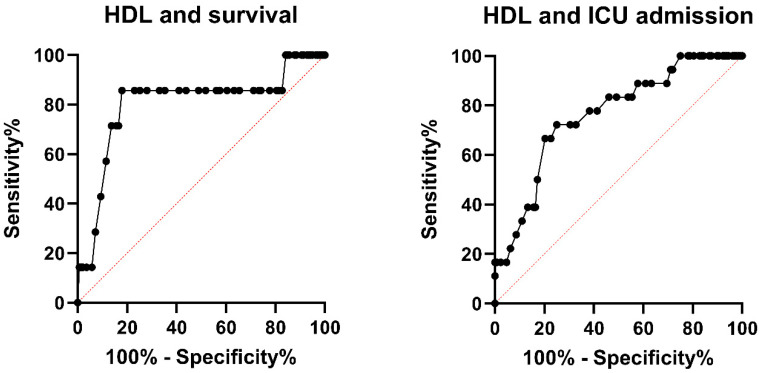
Receiver operator characteristic (ROC) analysis for HDL regarding intrahospital mortality and ICU admission among hospitalised male influenza patients. HDL measured within 48 h upon admission proved to be a prognostic marker regarding intrahospital mortality (AUC = 0.802; *p* = 0.003) as well as ICU admission (AUC = 0.762; *p* < 0.001). A threshold of HDL ≤ 25.5 mg/dL provides a sensitivity of 85.7% and a specificity of 82% regarding intrahospital mortality.

Logistic regression regarding intrahospital mortality with correction for age and the number of comorbidities was also calculated for other parameters, as indicated in Table 3. Apart from total cholesterol (OR = 0.975; *p* = 0.039), no other prognostic predictors were identified. HDL, however, remained a significant independent predictor for intrahospital mortality even after additional correction for initial CRP, HbA1c, admission blood glucose, and stress hyperglycaemia ratio (SHR) in respective separate models. Receiver operator characteristic (ROC) analysis was performed regarding the prognostic value of HDL regarding intrahospital mortality (AUC = 0.802; *p* = 0.003) as well as ICU admission (AUC = 0.762; *p* < 0.001), as shown in Figure 2 for male patients. Based on the ROC analysis, a threshold of HDL ≤ 25.5 mg/dL provides a sensitivity of 85.7% and a specificity of 82% regarding intrahospital mortality.

### 3.3. HDL and Inflammation Parameters

**Figure 3:**
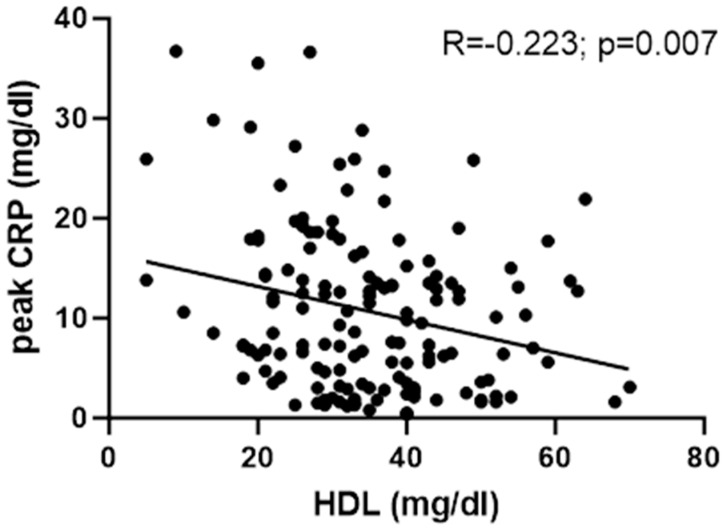
Correlation between HDL upon hospital admission (measured within 48 h) and peak CRP in hospitalised male influenza patients. There was a significant negative correlation between HDL and peak CRP (Spearman’s ρ = −0.223; *p* = 0.007). The correlation between HDL and peak CRP was very similar for all patients (Spearman’s ρ = −0.214; *p* = 0.007).

HDL concentrations were significantly negatively associated with peak CRP (Spearman’s ρ = −0.223 for male patients and ρ = −0.214 for all patients; *p* = 0.007 for both); see Figure 3. A significant negative association with peak CRP was also found for total cholesterol (ρ = −0.296; *p* < 0.001) and LDL (ρ = −0.302; *p* < 0.001).

Although total cholesterol (ρ = −0.294; *p* < 0.001) as well as LDL (ρ = −0.299; *p* < 0.001) was significantly negatively associated with the length of hospital stay, this association was not statistically significant for HDL and the length of hospital stay (ρ = −0.136; *p* = 0.102). Peak CRP as well as the length of hospital stay were not normally distributed, as assessed by Shapiro–Wilk test (*p* < 0.05).

## 4. Discussion

### 4.1. HDL as a Prognostic Marker in Influenza

In this study, we show that low HDL concentrations predict poor outcome and correlate with increased inflammation parameters in hospitalised influenza patients.

Lipoproteins exert important anti-inflammatory effects in bacterial infections and sepsis. Circulating bacterial pathogen-associated molecular pattern molecules (PAMPs) such as LPS and lipoteichoic acid (LTA) are bound by so called transfer proteins with subsequent incorporation by lipoproteins. This “lipid scavenging” presents an important mechanism of detoxification from PAMPs, thus reducing dysregulated innate immune response [8,19,20].

In clinical studies, low HDL is known to be associated with increased inflammatory response [8,10], and, in the general population, very low plasma HDL is associated with an increased risk of infectious disease [11]. This association on a population level also seems to be the case for SARS-CoV-2 infection [12,21]. However, to date, negative prognostic effects of low HDL in patients with infections have only been shown for bacterial infections and sepsis in ICU patients [13,14,15].

In this study, we show a negative correlation between HDL plasma concentrations and inflammation, as measured by the peak CRP in hospitalised influenza patients (Spearman’s ρ = −0.223; *p* = 0.007). This is consistent with previously reported data showing a negative association between HDL as well as LDL concentrations with inflammatory response in an experimental setting [8].

HDL values were significantly lower in patients who died during their hospital stay (median HDL 21 (IQR 19–25) mg/dL in deceased patients vs. 35 (IQR 28–44) mg/dL in survivors, *p* = 0.005), and ROC analysis showed a good prognostic value of HDL concentrations upon admission with an AUC of 0.802 (*p* = 0.003) regarding intrahospital mortality. We determined a ROC-informed HDL threshold of 25.5 mg/dL (sensitivity of 85.7% and specificity of 82% regarding intrahospital mortality), which matches very well with previously published data from a sepsis cohort in which an HDL threshold of 25.1 mg/dL was determined as a prognostic marker for adverse outcome [16].

After correction for patients’ age and the number of comorbidities (OR = 0.891; *p* = 0.009) and even after additional correction for initial CRP, HbA1c, and stress hyperglycaemia, HDL still proved to be a significant negative predictor for intrahospital mortality. When divided into groups according to HDL values, this prognostic impact became more pronounced—for every 10 mg/dL of higher HDL upon admission, the risk of intrahospital mortality was reduced by OR = 0.427 (*p* = 0.001). Similarly, HDL is a negative prognostic factor regarding ICU admission (OR = 0.911; *p* = 0.001).

This clearly shows that low HDL not only represents a risk factor for the increased risk of infections on a population level but that HDL is also a marker for prognosis upon admission not only in bacterial infections but also in influenza, an acute viral infection. So far, protective effects of HDL in infectious diseases regarding outcome have only been shown for bacterial infections and sepsis [13,14,15], where HDL values were found to outperform all other routine laboratory measures upon presentation in the emergency department including serum creatinine, platelet count, and lactate as a prognostic marker [16]. Regarding acute viral infections, HDL values measured before infection on a population-wide level were shown to be associated with COVID-19 infection rates as well as COVID-19-related mortality, as determined in a Spanish cohort [12,21].

However, our study is the first to show that HDL plasma concentrations upon admission are independently associated with mortality in influenza patients. Furthermore, this is the first study to show that the measurement of HDL upon hospital admission provides a marker for prognosis in acute viral infections.

### 4.2. The 2018/19 and 2019/20 Influenza Seasons and Limitations

In this study, we report data from 199 hospitalised patients at the St. John of God hospital Linz in Austria from the 2018/19 (n = 112) and 2019/20 (n = 87) influenza seasons. The mortality rate was 4.5% and 4.6% during the 2018/19 and the 2019/20 seasons, respectively. Thus, mortality was much lower compared to the mortality of 8.3% during the 2017/18 season at the St. John of God hospital Linz, an influenza season with high numbers of hospitalised patients and predominance of influenza type B, as previously reported by Obendorf et al. [17].

There are several limitations to our study. Firstly, there were only very few female influenza patients hospitalised at our institution (n = 26 in both seasons combined). This imbalance is due to the structure of regional hospital care, which leads to a distribution of male inpatients towards our institution and female patients to other hospitals. Since HDL concentrations were significantly higher among female patients, analysis was primarily performed exclusively in male patients to avoid bias. However, additional analysis in all patients revealed very similar results for all patients. Secondly, the number of patients included in the study was limited. We cannot provide repetitive measurements of HDL over the course of hospitalisation—previously published studies reported stable values over the course of hospitalisation in patients with sepsis [16]—nor can we provide evidence on the mechanistic processes behind the described association and the dynamic interplay between inflammation, antiviral immune response, and HDL. Bacterial superinfection and other complications are common in severe viral infections in hospitalised patients, and the cause of deterioration and death is usually multifactorial [22,23,24]. The mechanistic role of lipoproteins and HDL can thus not be examined in clinical studies like ours. However, the data clearly show that low HDL upon patients’ presentation in the emergency department is associated with increased inflammation and is a negative independent predictor regarding outcome in the collective of influenza patients.

Unfortunately, we cannot provide baseline HDL values of the patients included in this study from before the index infection. Furthermore, not all HDL measurements were performed at the exact same time point (HDL values were included when measured within 48 h upon admission). However, the measurement of HDL within the first 48 h reflects a real-world setting, and yet this study shows the prognostic value of HDL measurements in this setting.

To improve the prognostic assessment of patients presenting with infectious disease, HDL measurements may be a very valuable addition to standard laboratory parameters in the emergency department.

## 5. Conclusions

In conclusion, low HDL upon hospital admission is associated with increased inflammation and is an independent predictor for mortality in male patients with influenza A.

## Figures and Tables

**Table 1:** Characteristics of hospitalised patients with influenza stratified for intrahospital mortality. Data given as median (interquartile range) or n (% of the respective column). Data for metric parameters were not normally distributed except for total cholesterol as tested by the Kolmogorov–Smirnov test with Lilliefors correction (*p* > 0.05). *p*-values for metric values were calculated by Mann–Whitney U-test. Comparison for the number of comorbidities and malignant disease was performed using Fisher–Freeman–Halton test and Fisher’s exact test, respectively.

Parameters	Survived	Deceased	Overall	
Hospitalised patients	190	9	199	
Male (%)	164 (86.3)	9 (100)	173 (86.0)	
Female (%)	26 (13.7)	0 (0)	26 (13.1)	
Influenza A (%)	183 (96.3)	9 (100)	192 (96.5)	
Influenza B (%)	7 (3.7)	0 (0)	7 (3.5)	
ICU admission (%)	16 (8.4)	7 (77.8)	23 (11.6)	
Endotracheal intubation (%)	4 (2.1)	3 (33.3)	7 (3.5)	
Oseltamivir (%)	79 (73.8)	2 (40.0)	81 (72.3)	
Antibiotics (%)	118 (62.1)	9 (100)	127 (63.8)	
Lipid lowering therapy (%)	59 (31.1)	2 (22.2)	61 (30.7)	
Number of comorbidities (diabetes mellitus, arterial hypertension, kidney disease, coronary heart disease, COPD, and malignant disease)				
0 comorbidities (%)	56 (29.5)	1 (11.1)	57 (28.6)	*p* = 0.046
1 comorbidity (%)	43 (22.6)	0 (0)	43 (21.6)	
2 comorbidities (%)	40 (21.1)	5 (55.6)	45 (22.6)	
≥3 comorbidities (%)	51 (26.8)	3 (33.3)	54 (27.1)	
Malignant disease (%)	37 (19.5)	3 (33.3)	40 (20.1)	*p* = 0.388
BMI (kg/m^2^)	26.2 (23.6–29.7)	29.6 (24.5–32.5)	26.2 (23.8–29.8)	*p* = 0.358
Age (years)	69 (56–80)	72 (70–84)	70 (56–80)	*p* = 0.192
Body temperature (°C)	37.8 (36.8–38.6)	37.0 (36.6–37.9)	37.8 (36.8–38.6)	*p* = 0.288
CRP (initial, mg/dL)	3.5 (1.5–7.3)	6.9 (1.5–14.7)	3.5 (1.5–7.4)	*p* = 0.205
CRP (peak, mg/dL)	7.3 (3.0–13.3)	19.0 (13.8–27.2)	7.5 (3.2–13.8)	*p* < 0.001
Creatinine (initial, mg/dL)	1.05 (0.89–1.36)	1.06 (0.97–2.41)	1.06 (0.89–1.38)	*p* = 0.182
eGFR-MDRD (initial, ml/min/1.73 m^2^)	73 (54–90)	73 (29–78)	73 (54–90)	*p* = 0.219
Troponine-I (initial, ng/L)	12 (5–28)	59 (11–143)	13 (5–30)	*p* = 0.011
BNP (initial, pg/mL)	110 (31–280)	198 (105–361)	118 (35–290)	*p* = 0.137
HBA1c (%)	5.8 (5.4–6.5)	5.7 (5.3–5.8)	5.8 (5.4–6.5)	*p* = 0.523
Admission blood glucose (mg/dL)	118 (101–146)	128 (116–146)	118 (101–146)	*p* = 0.352
Stress hyperglycaemia ratio (SHR)	0.97 (0.86–1.20)	1.13 (1.07–1.39)	0.99 (0.86–1.20)	*p* = 0.112
Total cholesterol (mg/dL)	142 (112–165)	101 (92–115)	139 (111–164)	*p* = 0.027
Triglycerides (mg/dL)	95 (75–138)	106 (58–96)	96 (75–135)	*p* = 0.966
LDL (mg/dL)	82 (62–108)	62 (60–67)	82 (62–107)	*p* = 0.120
HDL (mg/dL)	35 (28–44)	21 (19–25)	34 (27–43)	*p* = 0.005
Duration of stay (days)	5 (3–10)	10 (6–16)	5 (3–10)	*p* = 0.084
Duration of stay ICU (days)	0 (0–0)	2 (1–10)	0 (0–0)	*p* < 0.001

**Table 2:** Characteristics of hospitalised patients with influenza stratified for ICU treatment. Data given as median (interquartile range) or n (% of the respective column). Data for metric parameters were not normally distributed except for total cholesterol as tested by the Kolmogorov–Smirnov test with Lilliefors correction (*p* > 0.05); *p*-values for metric values were calculated by Mann–Whitney U-test. Comparison for the number of comorbidities and malignant disease was performed using Fisher–Freeman–Halton test and Fisher’s exact test, respectively.

Parameters	No ICU Treatment	ICU Treatment	Overall	
Hospitalised patients	176	23	199	
Male (%)	151 (85.8)	22	173 (86.0)	
Female (%)	25 (14.2)	1 (4.3)	26 (13.1)	
Influenza A (%)	169 (96.0)	23 (100)	192 (96.5)	
Influenza B (%)	7 (4.0)	0 (0)	7 (3.5)	
Endotracheal intubation (%)	0 (0)	7 (30.4)	7 (3.5)	
Oseltamivir (%)	71 (71.2)	10 (66.7)	81 (72.3)	
Antibiotics (%)	107 (60.8)	20 (87.0)	127 (63.8)	
Lipid lowering therapy (%)	55 (31.3)	6 (26.1)	61 (30.7)	
Number of comorbidities (diabetes mellitus, arterial hypertension, kidney disease, coronary heart disease, COPD, and malignant disease)				
0 comorbidities (%)	52 (29.5)	5 (21.7)	57 (28.6)	*p* = 0.048
1 comorbidity (%)	42 (23.9)	1 (4.3)	43 (21.6)	
2 comorbidities (%)	38 (21.6)	7 (30.4)	45 (22.6)	
≥3 comorbidities (%)	44 (25.0)	10 (43.5)	54 (27.1)	
Malignant disease (%)	32 (18.2)	8 (34.8)	40 (20.1)	*p* = 0.092
BMI (kg/m^2^)	26.4 (23.9–29.7)	24.5 (22.7–32.5)	26.2 (23.8–29.8)	*p* = 0.672
Age (years)	70 (56–81)	69 (59–73)	70 (56–80)	*p* = 0.497
Body temperature (°C)	37.9 (36.9–38.6)	37.0 (36.4–38.1)	37.8 (36.8–38.6)	*p* = 0.056
CRP (initial, mg/dL)	3.5 (1.5–7.2)	3.6 (1.4–13.8)	3.5 (1.5–7.4)	*p* = 0.347
CRP (peak, mg/dL)	7.2 (3.0–12.8)	18.6 (13.3–25.9)	7.5 (3.2–13.8)	*p* < 0.001
Creatinine (initial, mg/dL)	1.05 (0.89–1.35)	1.15 (0.89–2.415)	1.06 (0.89–1.38)	*p* = 0.246
eGFR-MDRD (initial, ml/min/1.73 m^2^)	75 (55–90)	65 (31–90)	73 (54–90)	*p* = 0.334
Troponine-I (initial, ng/L)	12 (5–29)	15 (9–59)	13 (5–30)	*p* = 0.149
BNP (initial, pg/mL)	122 (33–291)	98 (58–198)	118 (35–290)	*p* = 0.829
HBA1c (%)	5.9 (5.5–6.5)	5.6 (5.1–5.9)	5.8 (5.4–6.5)	*p* = 0.014
Admission blood glucose (mg/dL)	117 (101–145)	132 (101–167)	118 (101–146)	*p* = 0.017
Stress hyperglycaemia ratio (SHR)	0.95 (0.86–1.18)	1.17 (0.97–1.39)	0.99 (0.86–1.20)	*p* = 0.017
Total cholesterol (mg/dL)	143 (116–167)	101 (92–133)	139 (111–164)	*p* < 0.001
Triglycerides (mg/dL)	95 (75–131)	106 (74–148)	96 (75–135)	*p* = 0.426
LDL (mg/dL)	82 (62–112)	62 (52–82)	82 (62–107)	*p* = 0.011
HDL (mg/dL)	35 (29–44)	26 (20–33)	34 (27–43)	*p* < 0.001
Duration of stay (days)	5 (3–8)	16 (10–30)	5 (3–10)	*p* < 0.001
Duration of stay ICU (days)	0 (0–0)	2 (2–10)	0 (0–0)	*p* < 0.001
Parameters	No ICU treatment	ICU treatment	Overall	

**Table 3:** Results of the logistic regression analysis regarding intrahospital mortality after adjustment for age and the number of comorbidities among hospitalised male influenza patients. HDL as well as total cholesterol proved to be significant predictors for intrahospital mortality after adjustment.

Parameters *	Odds Ratio (OR)	OR 95% Confidence Interval	*p*-Value
CRP	1.075	0.985–1.173	0.104
BMI	1.058	0.906–1.236	0.475
Body temperature	0.752	0.434–1.304	0.310
Creatinine initial	1.616	0.621–4.206	0.326
Glucose	1	0.989–1.012	0.985
HbA1c	0.672	0.275–1.643	0.384
SHR	4.785	0.451–50.773	0.194
LDL	0.970	0.932–1.011	0.146
HDL	0.891	0.817–0.972	0.009
Total cholesterol	0.975	0.952–0.999	0.039
Troponin-I	1	0.999–1.001	0.852
BNP	1	0.998–1.002	0.856

None of the other tested parameters were significant predictors for intrahospital mortality after adjustment. * All measurements were performed within 48 h upon admission.

## Data Availability

Data available on request due to privacy/ethical restrictions.

## Abbreviations

BNP	B-type natriuretic peptide
CRP	C-reactive protein
eGFR	Estimated glomerular filtration rate
HDL	High-density lipoprotein
IQR	Interquartile range
ICU	Intensive care unit
LDL	Low-density lipoprotein
LPS	Lipopolysaccharide
LTA	Lipoteichoic acid
MDRD	Modification of Diet in Renal Disease
OR	Odds ratio
PAMPs	Pathogen-associated molecular patterns
ROC	Receiver operator characteristic
SHR	Stress hyperglycaemia ratio
